# Gastric Mucosa-Associated Lymphoid Tissue Lymphomas Diagnosed by Jumbo Biopsy Using Endoscopic Submucosal Dissection: A Case Report

**DOI:** 10.3389/fmed.2021.668531

**Published:** 2021-06-07

**Authors:** Jian Han, Jun Wang, Hua-ping Xie

**Affiliations:** ^1^Department of Gastroenterology, Tongji Hospital of Tongji Medical College, Huazhong University of Science and Technology, Wuhan, China; ^2^Department of Pathology, Tongji Hospital of Tongji Medical College, Huazhong University of Science and Technology, Wuhan, China

**Keywords:** mucosa-associated lymphoid tissue lymphoma, endoscopic submucosal dissection, flow cytometry, jumbo biopsy, stomach

## Abstract

The stomach is the most common primary site of mucosa-associated lymphoid tissue (MALT) lymphoma, and sometimes the histopathological diagnosis is particularly difficult. An endoscopic forceps biopsy is the primary diagnostic test, but false negative results are very common. Therefore, a jumbo biopsy is essential for accurate diagnosis of clinically suspected cases. Here we diagnosed two cases of gastric MALT lymphomas using endoscopic submucosal dissection (ESD). The first patient was suspected of gastric lymphoma at the first endoscopic forceps biopsy, but the second endoscopic forceps biopsy showed chronic inflammation. The second patient was also firstly diagnosed with chronic inflammation by endoscopic forceps biopsy. Both cases were finally confirmed with the diagnosis of gastric MALT lymphoma by jumbo biopsy using ESD. The application of ESD can provide a new diagnostic strategy for clinically suspicious cases of gastric MALT lymphoma with negative endoscopic forceps biopsy.

## Introduction

MALT lymphoma, classified as an indolent B-cell non-Hodgkin lymphoma, arises in extra-nodal sites from the malignant transformation of B lymphocytes that are mainly triggered by infection or autoimmune process (1). Although they can exist in different organs such as the salivary gland, thyroid gland, breast, lung, bladder, skin, and orbit, MALT lymphomas are most frequently detected in the gastrointestinal tract (2). The most frequently affected organ is the stomach, where MALT lymphoma is incontrovertibly associated with chronic gastritis induced by a microbial pathogen, Helicobacter pylori (3). The incidence of gastric MALT lymphoma is increasing, but the diagnosis is difficult (4). Most patients are asymptomatic or complain of non-specific symptoms (4). Gastric MALT lymphoma shows a variable endoscopic appearance, including erosion, erythema, discoloration, atrophy, ulcer, and subepithelial lesion (5). As the endoscopic features of gastric MALT lymphoma are variable and non-specific, the possibility of this condition may be overlooked (4). An endoscopic forceps biopsy is the primary diagnostic test, but false negative results are possible (4). Therefore, clinical suspicion and jumbo biopsy are essential for accurate diagnosis (5). ESD may provide a new strategy to acquire large tissue samples for jumbo biopsy as it is an emerging method to cure early gastrointestinal carcinomas and submucosal tumors (6, 7). Here we report two cases of gastric MALT lymphomas diagnosed by jumbo biopsy using ESD. The first patient was a 36-year-old female, and she was admitted to our hospital because of stomachache, nausea, and vomiting. Physical examination showed no pathological signs. The blood routine test showed mild leukopenia (white blood cell 3.08^*^109/L) and moderate anemia (Hemoglobulin 73 g/L). The fecal routine test and occult blood test were normal. The blood biochemistry test, tumor markers and the urine routine test were all in normal ranges. The second patient was a 53-year-old female, and she was admitted to our hospital because of abdominal discomfort. Physical examination showed no pathological signs. The blood routine test showed mild thrombocytopenia (platelet 118^*^109/L). The blood biochemistry test, the fecal routine test and occult blood test were all in normal ranges.

## Case Description

**Figure 1:**
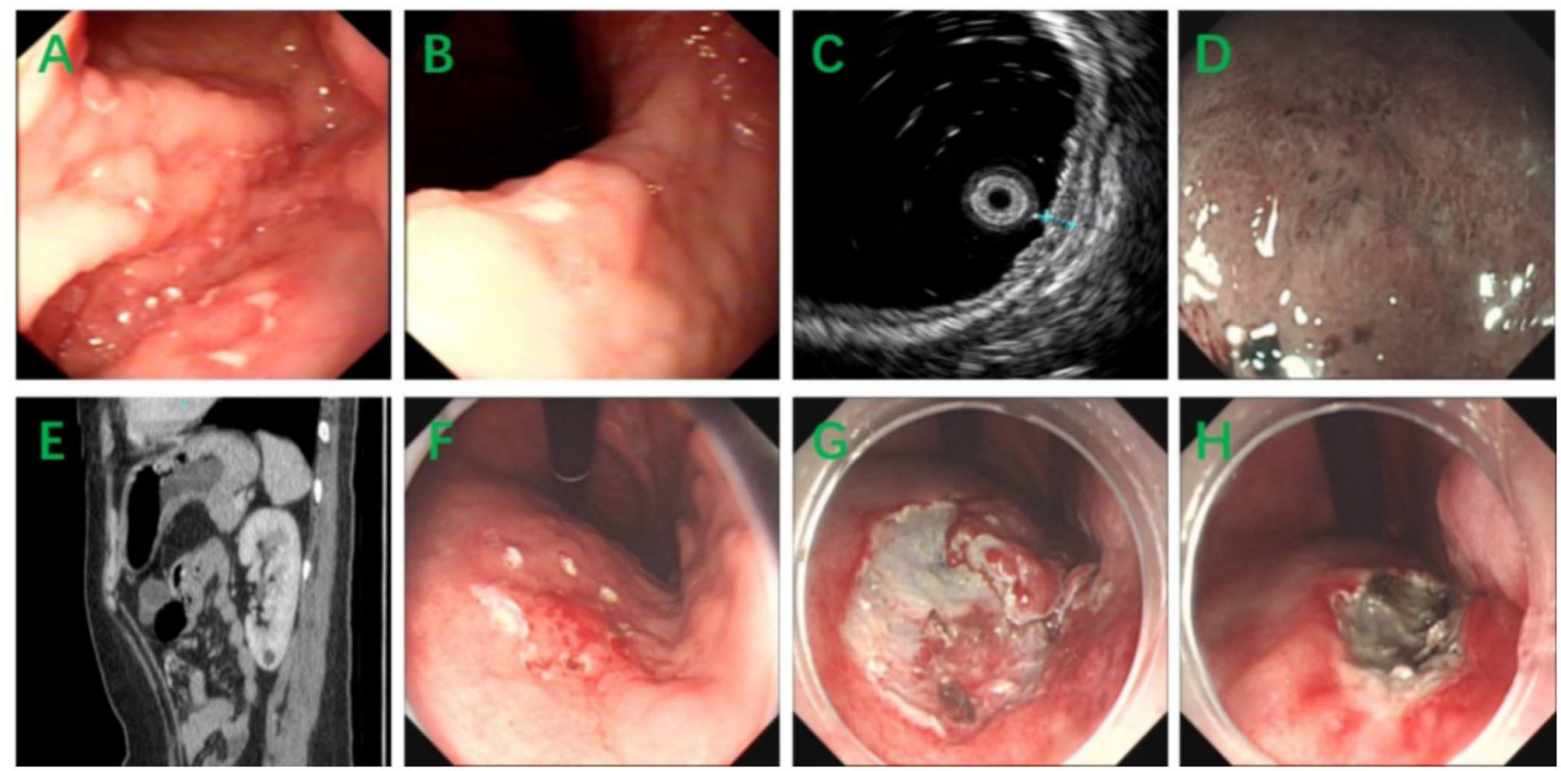
**(A,B)** Gastroscopy revealed multiple erosion and ulcer in gastric body and gastric angle. **(C)** EUS revealed hypoechoic thickening of the mucosa layer. **(D)** ME-NBI showed irregular marginal crypt epithelium and subepithelial capillary network. **(E)** Abdominal computed tomography showed thickening of the wall of gastric body and gastric fundus and mild enhancement, and the surrounding lymph nodes were enlarged. **(F–H)** The procedure of ESD.

**Figure 2:**
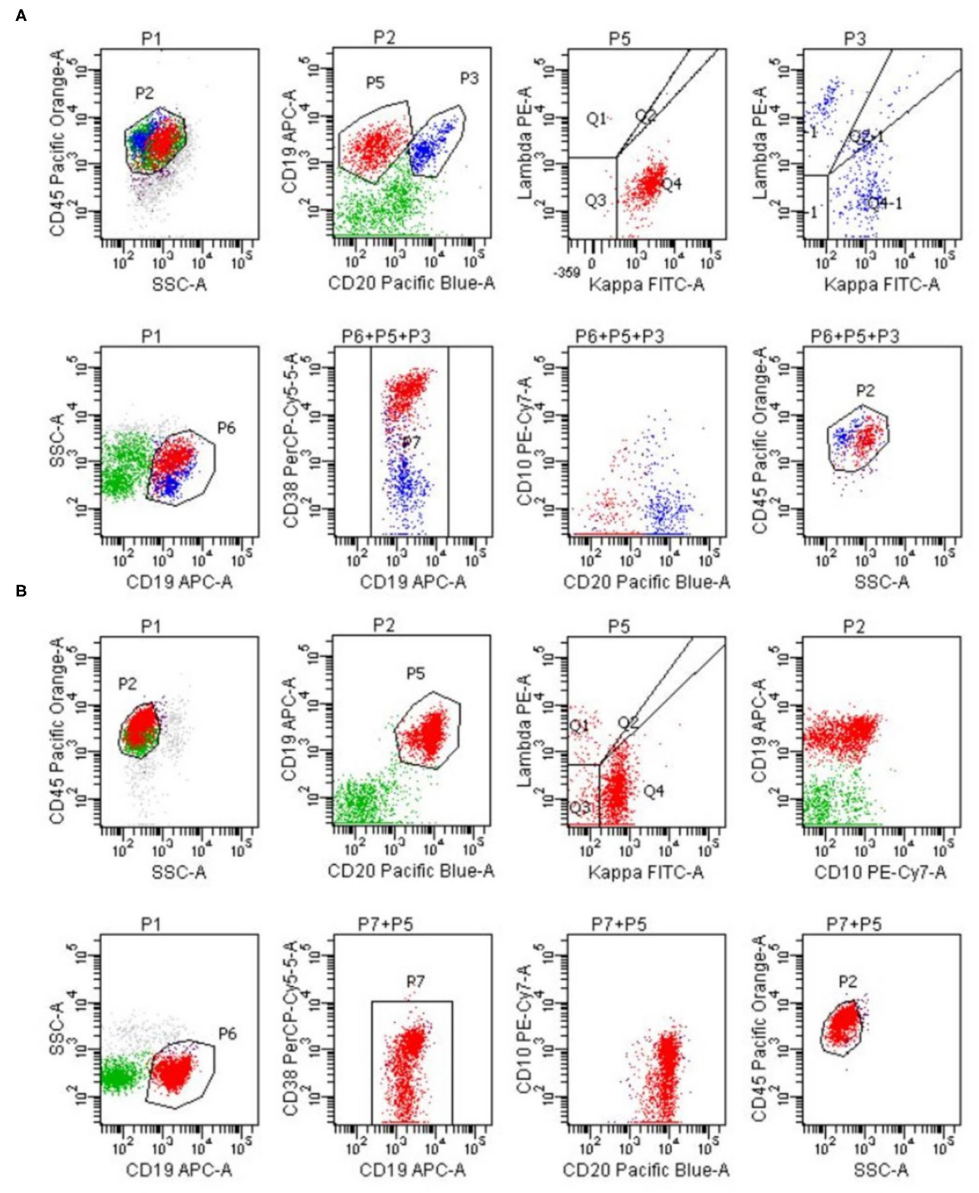
**(A)** The flow cytometry showed that ~23.9% of all the lymphocytes (red cell population) expressed CD19, CD20, CD38, kappa, and did not express lambda, CD10. **(B)** The flow cytometry of the ESD sample showed that ~39.5% of all the lymphocytes (red cell population) expressed CD19, CD20, kappa, CD38 (partial), and did not express lambda, CD10.

**Figure 3:**
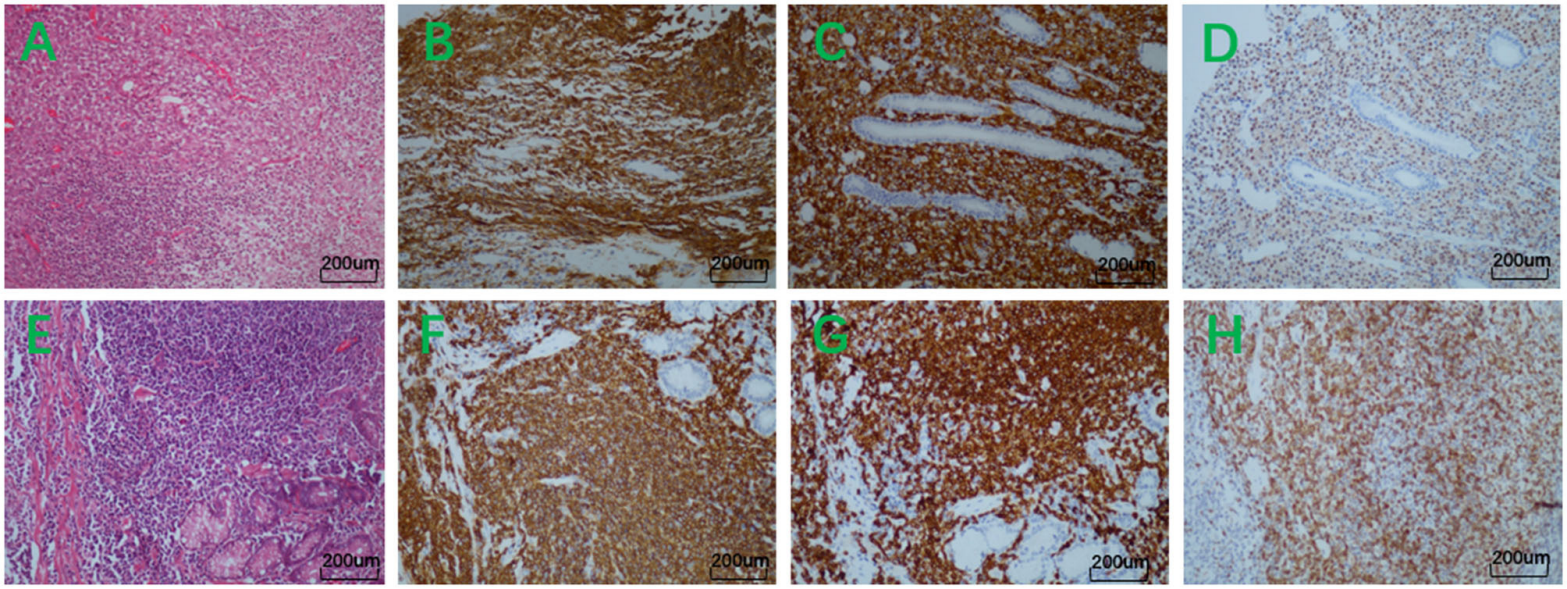
**(A)** Hematoxylin-eosin staining × 200. **(B)** Immunohistochemistry showed positive reactivity for CD20. **(C)** Immunohistochemistry showed positive reactivity for CD79a. **(D)** Immunohistochemistry showed partially positive reactivity for Mum-1. **(E)** Hematoxylin-eosin staining × 200. **(F)** Immunohistochemistry showed positive reactivity for CD20. **(G)** Immunohistochemistry showed positive reactivity for CD79a. **(H)** Immunohistochemistry showed positive reactivity for Bcl-2.

The first patient was a 36-year-old female, and she was admitted to our hospital because of stomachache, nausea, and vomiting. Physical examination showed no pathological signs. The blood routine test showed mild leukopenia (white blood cell 3.08^*^109/L) and moderate anemia (Hemoglobulin 73 g/L). The fecal routine test and occult blood test were normal. The blood biochemistry test, tumor markers, and the urine routine test were all in normal ranges. Gastroscopy revealed multiple erosion and ulcer in gastric body and gastric angle (Figures 1A,B) and biopsy showed atypical lymphocytes and gastric lymphoma was suspected. The ^13^C urea breath test was negative for helicobacter pylori. Endoscopic ultrasonography (EUS) revealed hypoechoic thickening of the mucosa layer (Figure 1C) and magnifying endoscopy with narrow band imaging (ME-NBI) showed irregular marginal crypt epithelium and subepithelial capillary network (Figure 1D), but the second biopsy showed chronic inflammation. Abdominal computed tomography showed thickening of the wall of gastric body and gastric fundus and mild enhancement, and the surrounding lymph nodes were enlarged (Figure 1E). Based on the endoscopic findings, imaging features and repeat insignificant biopsy results, a diagnose of gastric MALT was suspicious and therefore we performed ESD for jumbo biopsy (Figures 1F–H). After ESD, no adverse and unanticipated events happened. The flow cytometry of the ESD sample showed that ~23.9% of all the lymphocytes (red cell population) expressed CD19, CD20, CD38, kappa, and did not express lambda, CD10, which were considered as abnormal monoclonal B lymphocytes with plasma cell differentiation or lymphoid plasma cells (Figure 2A). Therefore, a diagnosis of gastric MALT lymphoma was suspected. The histopathological examination of the ESD sample confirmed the diagnosis of a gastric MALT lymphoma with plasma cell differentiation, with diffuse infiltration of small-sized lymphoid cells, which were positive for CD20, PAX-5, Mum-1 (partial), Bcl-2 (partial), CD21 (partial), CD79a, but negative for CD3, CD5, CD10, CD43, Bcl-6, SOX11, and cyclin D1 (Figures 3A–D). The Ki-67 index was 2%.

**Figure 4:**
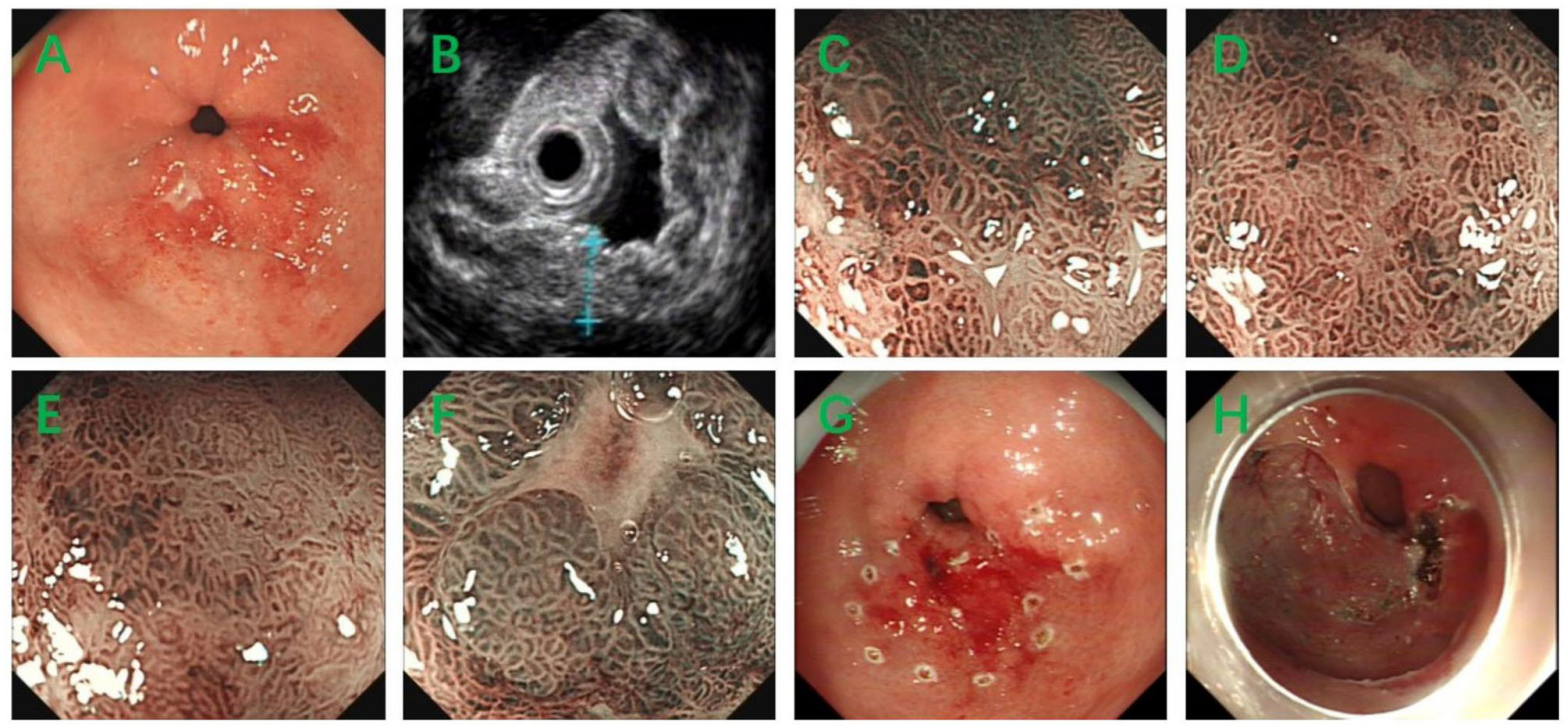
**(A)** Gastroscopy revealed erosion and ulcer in greater curvature of gastric antrum. **(B)** EUS revealed a hypoechoic lesion from mucosa layer, muscularis mucosa layer, and submucosa layer. **(C–F)** ME-NBI showed irregular marginal crypt epithelium and subepithelial capillary network. **(G,H)** The procedure of ESD.

The second patient was a 53-year-old female, and she was admitted to our hospital because of abdominal discomfort. Physical examination showed no pathological signs. The blood routine test showed mild thrombocytopenia (platelet 118^*^109/L). The blood biochemistry test, the fecal routine test and occult blood test were all in normal ranges. Gastroscopy revealed erosion and ulcer in greater curvature of gastric antrum (Figure 4A) and biopsy showed chronic inflammation. The ^13^C urea breath test was negative for helicobacter pylori. EUS revealed a hypoechoic lesion from mucosa layer, muscularis mucosa layer and submucosa layer (Figure 4B) and ME-NBI showed irregular marginal crypt epithelium and subepithelial capillary network (Figures 4C–F), but the second biopsy showed chronic inflammation and intestinal metaplasia. Based on the endoscopic findings and repeat insignificant biopsy results, we performed ESD for jumbo biopsy (Figures 4G,H). After ESD, no adverse and unanticipated events happened. The flow cytometry of the ESD sample showed that ~39.5% of all the lymphocytes (red cell population) expressed CD19, CD20, kappa, CD38 (partial), and did not express lambda, CD10, which were considered as abnormal monoclonal B lymphocytes (Figure 2B). Therefore, a diagnosis of gastric B-cell lymphoma was suspected. The histopathological examination of the ESD sample confirmed the diagnosis of a gastric MALT lymphoma, with diffuse infiltration of small-sized lymphoid cells, which were positive for CD20, CD79a, PAX-5, Bcl-2, Mum-1 (partial), CD21 (partial), CD23 (partial), kappa (partial), and lambda (partial), but negative for CD3, CD5, CD10, CD43, Bcl-6, cyclin D1, c-myc, SOX11, and p53 (Figures 3E–H). The Ki-67 index was 5%.

## Discussion

The incidence of gastric MALT lymphoma is increasing, but the diagnosis is difficult (4). Most patients are asymptomatic or complain of nonspecific symptoms (4). The endoscopic features of gastric MALT lymphoma can be classified into exophytic, ulcero-infiltrative, and superficial types; ulcero-infiltrative type is the most common, accounting for ~40–50% of all cases (4). As the endoscopic features of gastric MALT lymphoma are variable and non-specific, the possibility of this condition may be overlooked during gastroscopy (4). Endoscopic biopsy using forceps and histopathologic examination are the most basic tests for diagnosis of gastric MALT lymphoma (4). However, false negative results may be possible because the tumor cells of gastric MALT lymphoma originate from the deep mucosa or submucosa and grow without destroying the foveolar gland, which is the basic structure of the mucosal surface (4). Therefore, clinical suspicion and jumbo biopsy are essential for accurate diagnosis (5). More invasive tissue biopsy such as endoscopic mucosal resection (EMR) or ESD may be required if the diagnosis is not confirmed by routine endoscopic biopsy (8–10). ESD may provide a new strategy to acquire large tissue samples for jumbo biopsy as it is an emerging method to cure early gastrointestinal carcinomas and submucosal tumors (6, 7). Compared to EMR, ESD is superior because it allows en bloc resection and accurate histological examination (11). This case report focused on an important clinical issue and offered a potential way to increase diagnostic accuracy. This report showed a comprehensive evaluation of two cases including complete history and clinical examination (including helicobacter pylori and immunohistochemistry) and comparisons with EUS and ME-NBI. The disadvantage is that ESD is more invasive than biopsy, and there is a risk of bleeding and perforation, and the cost is higher. ESD is only suitable for patients who cannot be diagnosed with repeated biopsy. In conclusion, we report two cases of gastric MALT lymphomas with ulcero-infiltrative type diagnosed by jumbo biopsy using ESD. ESD may be recommended as a reasonable option for the diagnosis of gastric MALT lymphomas in properly selected cases in which adequate tissue samples are difficult to obtain, as it is effective to acquire large specimen and minimally invasive. More data are required to provide better insights for this disease.

## Data Availability Statement

The raw data supporting the conclusions of this article will be made available by the authors, without undue reservation.

## Ethics Statement

Ethical review and approval was not required for the study on human participants in accordance with the local legislation and institutional requirements. The patients/participants provided their written informed consent to participate in this study. Written informed consent was obtained from the individual(s) for the publication of any potentially identifiable images or data included in this article.

## Author Contributions

JH and H-pX designed the study. JW performed the pathologic analysis. JH wrote the original draft. H-pX was responsible for the revision of the manuscript and performed ESD. All authors read and approved the manuscript.

## Conflict of Interest

The authors declare that the research was conducted in the absence of any commercial or financial relationships that could be construed as a potential conflict of interest.
